# Broken dreams of a better life in Sweden: Thai women’s lived experiences of intimate partner violence by Swedish men in international marriages

**DOI:** 10.1080/16549716.2018.1496889

**Published:** 2018-08-03

**Authors:** Weerati Pongthippat, Mehrdad Darvishpour, Jureerat Kijsomporn, Gunnel Östlund

**Affiliations:** a School of Health, Care and Social Welfare, Mälardalen University, Eskilstuna, Sweden; b Mental Health and Psychiatric Nursing Department, Boromarajonani College of Nursing Udon Thani, Udon Thani, Thailand; c Praboromrajchanok Institute for Health Workforce Development, Ministry of Public Health, Nonthaburi, Thailand

**Keywords:** Economic migration, domestic violence, mental illness, Asian wives, female migrants

## Abstract

**Background**: Intimate partner violence by men against women has detrimental effects on equality, health and integration. Migrated and ‘imported’ wives experience an increased risk of intimate partner violence.

**Objectives**: The purpose of this study was to explore Thai immigrant women’s lived experiences of intimate partner violence in Sweden.

**Method**: Semi-structured interviews based on the critical incident technique with specific questions about experiences of male-to-female intimate partner violence were used to collect data. The participants were Thai immigrant women who had lived in Sweden for more than five years. Qualitative content analysis was used to identify patterns and variations in the transcribed data material.

**Results**: Eighteen interviewees reported psychological, physical, economic and/or sexual violence in their international marriages. These Thai women described being faithful and silent and reliable housewives. However, this did not keep them from being replaced and losing dignity as a result of intimate partner violence, including experiencing broken dreams and deception. Although their dreams were broken, they did not give up their efforts to achieve better lives in Sweden.

**Conclusions**: The vulnerability of imported wives in international marriages needs to be further recognised by health and welfare agencies in Sweden, as elsewhere, to ensure that these women have equal access to human rights, welfare and health as other citizens. From a health promotion perspective, home-based health check-ups are needed to stop the exploitation of imported wives. In Thailand, information and education about the unrecognised negative conditions of the Mia farang role (Imported wife role) need to be disseminated.

## Background

The Thai women who are the focus of the present paper are termed ‘imported wives’ because they have moved to a new country as part of an in international marriage or partnership. The largest proportion of ‘import marriages’ occur between Swedish men and women from Southeast Asia [1,2]. Imported wives’ marriages tend to be more unequal than other partnerships [3,6] and often include dependency and vulnerability on the woman’s part. Swedish-born males in international marriages are generally older and less well educated than the average married male [3–7].

Statistics show that Thai women are most often granted residence permits based on family factors, mainly through marriage (93%), and few emigrate for work, educational studies or humanitarian reasons [3]. The number of Thai women arriving in Sweden for marital purposes increased dramatically in recent years, from 17,099 in 2009 to 36,974 in 2013 [3,8]. The number of Thai women who were registered as living with a Swedish man was 16,000 in December 2017 [3].

Thai women tend to marry foreigners to improve their living conditions and lifestyle [9]. Additionally, Thai family and relatives request financial support from these imported wives, based on the needs and poverty levels of Thai families [10–14]. Thai women are active agents in their migration process, but the transition to another country often results in unexpected problems in the new country [8]. Adaptation to a new country is a process of personal change and adjustment [15]. Emigrants often carry distress with them from one nation or culture to the new one. In effect, migration is a stressful life event that may be associated with subsequent marital instability, which, in turn, influences immigrant women’s health [16,17]. Moreover, imported wives increase the flow of women abandoning their family and children and relocating their home, which opens up new areas of so-called transnational spaces within which perpetrators can perform new forms of violence against women, often with impunity [18].

Violence against women has been identified as a major public health and human rights problem throughout the world [19–21]. The World Health Organization (WHO) emphasises that violence by men towards women is a significant health problem affecting one in three women globally [19]. Addressing violence against women is critical for both health and family reasons [22–25]. Research has shown that male intimate partner violence against women can increase when the husband experiences a loss of power and lack of control in addition to socioeconomic poverty; moreover, women’s increased educational level and power position might provoke men [26]. Male pathologies are often still used to explain intimate partner violence against women; men who commit such acts are likely to be insecure due to increased distress, anger/hostility, personality disorders, alcohol problems and lack of secure attachments [27]. In situations where men experience a lack of control within their partnership, they may react violently as a means of exerting control over their partner [28]. Research has shown that powerless men exhibit violence to a greater extent [29] and that men might feel empowered as part of a group but not individually [26,30].

To understand male intimate partner violence against women in the globalised world, an ecological model of social-level factors has been developed that focuses on the individual, the relationship, the community, the social context and global exchange [30]. This ecological model focuses on violence against women worldwide, but there is not a specific emphasis on international marriages. In the present study, we wanted to learn more about the situation of Thai imported wives in Sweden with a particular focus on male intimate partner violence (IPV) against women since few studies have been conducted on this topic [31].

Previous research has shown that the lifetime prevalence of IPV among Thai women residing in Sweden was 22% and that exposure was also significantly related to poor mental health [31]. Fernbrant et al. (2014) concluded that obstacles to integration for Thai immigrant women were social isolation and mental health problems, and they suggested social trust as a resilience factor. In a follow-up study exploring the social process of migration among Thai imported wives, it was found that self-strength and social relationships promoted these women’s well-being and ability to leave unhealthy relationships [32]. However, more knowledge is needed to make the health situation of Thai imported wives visible and manageable through research in Sweden and elsewhere. Therefore, the present study aimed to explore Thai immigrant women’s lived experiences of intimate partner violence by Swedish men.

## Methods

This study had a qualitative interview design. The interview guide was semi-structured with questions based on the critical incident technique (CIT) [33]. The CIT methodology was designed to categorise the difficult situations that people experience and the consequences of these critical incidents [34]. The CIT can be understood as a phenomenological approach [35,36] appropriate for studying human lived experiences in specific situations. Moreover, by using described appraisals of behaviour, the CIT can explore experienced social, emotional and moral dilemmas [37,38] to elucidate participants’ perspectives on situations. In health and welfare research, the CIT has been used as a data collection method to help the researcher focus on specific situations in everyday life to collect high-quality data, but the CIT includes no specific methodology for data analysis.

### Description of the participants

The participants in the present project were recruited according to the following criteria: (a) born in Thailand and self-identified as Thai, (b) lived in Sweden for at least five years, and (c) currently or previously married to a native-born Swedish man.

To find a group of participants with sufficiently varied experiences, three different sizes of cities were included in areas close to the capital of Sweden. The data collection sources were three Thai cultural associations since they engage a large number of Thai immigrants living in Sweden. The use of Thai cultural associations to support data collection was our second choice; our first choice, using the Central Bureau of Statistics to conduct qualitative interviews, was too expensive due to the small number of interviews. In the present project, 60 women were invited to participate through invitation by 3 chairpersons of these Thai cultural organizations, based on inclusion criteria that were determined beforehand. An invitation letter was sent to the chairpersons of Thai cultural associations describing the research project and asking about the possibility of contacting Thai women to request their participation in the research. Two of these Thai women missed repeated appointments, one declined participation because of discomfort with sharing her experiences and one was forbidden to participate by her partner. Finally, 40 women were interviewed in the present project. In exploring the women’s experiences of intimate partner violence as part of the planned project aim,we did not know whether this research question could be treated as a separate study. However, after having received 14–15 participants who reported IPV experiences, the data were moving towards saturation and we decided to treat this dataset as a seperate sample. In total, 18 of the 40 interviewees in the project reported IPV. The participants had lived in Sweden for an average of 19 years at the time of the interviews, most of them had children, most had 12 years or more of education, and half originated from northeastern Thailand (Table 1).

**Table 1.** Sociodemographic data of the 18 Thai immigrant women who reported experiences of domestic violence in Sweden, 2016.

Sociodemographic characteristics			*N *= 18	Mean years
Age at the interview			35–68	52
Age upon arrival in Sweden			23–50	33
Length of time living in Sweden			6–43	19
Region of origin in Thailand*		Northeastern (Isan)	9	
		North	1	
		South	1	
		Central	7	
Marital status		Cohabiting	8	
		Living apart	5	
		Divorced	5	
Children				
	Thai women who have children		16	
	Thai women who have no children		2	
Number of children				
	Thai children		13	
	Half Thai- half Swedish		10	
Location where children currently reside				
	Half Thai- half Swedish living in Sweden		10	
	Thai children living in Thailand		7	
	Thai children living in Sweden		6	
Education				
(1) Illiterate			3	
(2) Primary school (6 years)			2	
(3) High school (12 years)			2	
(4) College (Courses at bachelor level)			5	
(5) Bachelor’s degree			6	

* Birth province/part of Thailand where the participants lived for a significant time

### The interview

The interview questions were first pilot tested to appraise their functionality and to test the interview procedure; following the pilot testing, small corrections were made to the questions [39]. During the phone call from the interviewer, the time, date and location of the interview were decided. The Thai immigrant women chose the location of the interview, which took place at their home, their workplace, a Thai temple or the university. The possible participants received verbal information in Thai regarding the objective of the study, both during the call and at the time of the interview, emphasising that participation was voluntary and that they had the right to withdraw at any time before publication of the results.

During the interviews, the Thai immigrant women were asked to share their experiences and were encouraged to provide and expand on detailed information based on the described specific situation. All the interviews started with the following central question: ‘How do you experience everyday life?’ and then continued with guided questions; for example, ‘Could you please describe how your everyday life works in term of being a “Thai-Swedish wife” or Mia farang or Panya farang?’ However, if the question of male intimate partner violence against the woman had not been mentioned earlier in the interview by the interviewees themselves in terms of critical situations, the following questions were posed at the end of the interview: ‘Do you recognise that there are four forms of abusive situations, such as physical, psychological, economic and sexual violence?’ and ‘Have you experienced any threats or abusive situations since coming here?’

The length of the interviews depended on each participant and ranged from 50 to 90 minutes, and all the interviews were conducted in the language of the Thai woman’s choice: Thai, the Isan local dialect or Thai mixed with English. Data were collected from 11 July 2016 to 12 December 2016. The interviews were audiotaped and transcribed verbatim into Thai by the first author.

### Data analysis

Qualitative content analysis was used when interpreting the collected data [40]. The transcriptions were read several times to identify passages that included meaning units that were relevant to the study aim. These meaning units were condensed and labelled with codes that were later grouped into subcategories and categories. To validate the preliminary results, the other bilingual co-author (JK) read the transcribed interviews in Thai and suggested interpretations. The meaning units were translated from Thai into English, and the analysis was further discussed among the research team to ensure a close connection among the original transcripts, the identified meaning units, and the subcategories and categories when framing the results. An example of our data analysis is presented in Table 2.

**Table 2.** Examples of content analysis: Thai immigrant women’s reported experiences of psychological violence from interviews in Sweden, 2016.

	Interview #1	Interview #2
Meaning units	We have been living together for seven years and have two sons, six and one-and-a-half years old. He was a great man in the beginning, but soon into the marriage I realized he was immature … he was [taking] fewer responsibilities […] playing [video] games, watching TV, or socializing with his friends but disliked cleaning or taking care of the kids… He liked to call his mother to help […] I was very stressed when he started a new job. He worked night shifts and sometimes in the early morning […] He complained about his job. He was annoyed and angry with everything. We quarrelled; he was mad and angry at the kids, shouting and saying bad words to us, throwing things on the floor […] I had warned that I might divorce him if he did not improve his behaviour	His income was less than mine, and we had many arguments when I visited my family or gave money to my family … he was jealous and never understood that in the Thai culture, we have to take care of family […] My ex-Swedish husband was lazy, lacked responsibility and enthusiasm and did not do anything (household chores). I did everything by myself. I climbed on the ladder to change lightbulbs, made repairs when the water did not flow […] I was tired and gave up on him. He never hit me, but he used evil words and hurt my feelings. […] Finally, I asked him for a divorce
Condensed meaning units	Swedish husband does not help with or take care of children; he just spends time on his own	Thai wife wants Swedish husband to help out in the household, understand Thai culture and share family responsibilities. Husband withdraws from responsibilities and quarrels
Codes	Married to a mum’s boy	Cultural differences
Subcategory	Family responsibilities	
Category	Reliable housewife	

### Ethical considerations

All informed consent forms were written in Thai and verbally explained, and the participants were asked to read the consent form carefully before signing their consent to become a research participant [41]. They all understood that participation was voluntary, and they had received information about the objectives of the study. Confidentiality, anonymity and the right to withdraw at any time before publication of the results were also discussed and guaranteed.

After the interview, the interviewer asked the interviewee whether the questions in the interview had made her feel uncomfortable and whether she knew who to turn to if that was the case. The interviewer also reminded the the interviewee that an address for health care support was listed in the consent form she had just received.

The participants’ integrity was preserved by using fictional names in the results section and by withdrawing specific details (for example, in quotes) that might jeopardise their integrity; however, the women’s age, educational level and type of work in Sweden were included. Additionally, approval for the project was received by the Regional Ethical Review Board in Uppsala, number 2016/542.

## Results

The intimate partner violence by Swedish men reported in the interviews showed that the immigrant women had experienced not only psychological violence in their family life in particular, but also a combination of physical, economic and sexual violence (Table 3). The interviewed Thai women described in detail their experiences with how they handled the domestic violence by acting *faithful and silent* and being a *reliable housewife*. However, this did not keep them from *being replaced and losing dignity* through experiencing *broken dreams and deception*. These main categories (written in italics) were interpreted through the content analysis in the present study (Table 4).

**Table 3.** Thai imported wives’ experiences of domestic violence from interviews in Sweden, 2016.

Types of domestic violence	*N *= 18
Psychological violence (exclusively)	9
Physical and psychological violence	6
Physical, psychological and economic violence	1
Psychological and sexual violence	1
Psychological, economic and sexual violence	1

**Table 4.** Domestic violence as experienced by Thai immigrant women from interviews in Sweden, 2016.

Categories	Subcategories	Codes
Faithful and silent	Violence as a family issue	Violence as part of marriage
		Violence is normal
		Violent treatment as embarrassment
	Endure and be patient	Dust-up
		Drinking and using drugs
		Violent treatment
Reliable housewife	Family responsibilities	Married to a mum’s boy
	Engagement and consideration	Unequal relationship
		Giving without getting
Being replaced and losing dignity	Not good enough	Involved with other women
		Being asked for a divorce
	Homeless and heartless	Break-up feelings and mental problems
		Losing home, business and self-esteem
Broken dreams and deception	My children and others’ kids	Native Thai children
	Expectations of Mia farang	Swedish ex-wife and mother-in-law
		Exploitation; sex and housemaid

### Faithful and silent

From the interviewees’ perspective, male intimate partner violence in family life was something that a wife had to endure. It was almost normal, and you had to be patient, according to how these immigrant women without citizenship thought. Being patient was also the advice given by Thai female friends in Sweden. A common description of the violence that the interviewed women endured involved the men becoming hostile and angry, using insulting terms and threatening the women; in some cases, the abuse escalated to physical violence:

Many times, he liked to drink and hang around with friends. He would leave me alone with my little daughter, and when he came back home, he would be angry with everything, and he pushed me and beat me. I fell and got abrasive wounds on my body. There were red and bluish-purple colours on my wounds, and I did not go out for a few days. It was family life, and I thought it was simple and normal **[**just a small thing in the family**]**, and **[**it made me silent**]**. (Tangkua, 50, illiterate, cleaner)

The intimate partner violence, particularly when combined with drug and alcohol abuse, made family life almost intolerable. Nonetheless, some couples were in love, especially at the beginning of their international marriages, after cohabiting for a while or even after living together for several years as a married couple. Thai women reported they fulfilled wives’ duties, such as being faithful and patiently enduring their Swedish husbands’/partners’ temper and violence. Enduring and being patient for at least two years was also advice received from Thai female friends in Sweden.

### Reliable housewife

The intimate partner violence by the men against the interviewed women sometimes included economic violence, such as having to feed the children and the family without sufficient economic resources and being given an inadequate amount of household cash each month. According to the imported wives, intimate partner violence was closely related to their husbands’ jealousy, paranoia, laziness, lack of enthusiasm, and withdrawal from family responsibilities. Some husbands had abandoned all adult responsibilities and never took care of the children or did any housework. In one of the interviews, the woman explained that even if the children were injured or sick with pneumonia, her husband went out to spend money and socialise with friends instead of caring for the family:

Three weeks after I entered Sweden, I met and married [a] Swedish man. We have been living together for seven years and have two sons, six and one-and-a-half years old. He was a great man in the beginning, but soon into the marriage, I realised he was immature; he was [taking] on fewer responsibilities […] playing [video] games, watching TV, or socialising with his friends. He disliked cleaning or taking care of the kids and shouted at them … often he called his mother to help. […] I had warned him that I might divorce him if he did not improve his behaviour; now [he] just makes a [big] deal about having sex. (Chaba, 35, Thai, partially finished college degree, restaurant worker)

Most of the Swedish husbands did not understand or want to learn about Thai Buddhism and cultural traditions, and from the women’s point of view this was almost a betrayal because family responsibilities and cultural habits are closely connected in the Thai community. Moreover, the interviewed women reported that their husbands did not accept them sending (self-earned) money to their parents and complained about it, although the women explained that a crucial aspect of Thai traditional norms and values is offspring showing respect and trust towards the parents who raised and supported them.

### Being replaced and losing dignity

Some of these international marriages ended in divorce or break-up. The interviewed women described that losing their husbands in a foreign country where the male partner was the chief link to society was a very stressful experience that affected their health and well-being, especially if the Thai woman was illiterate in both the foreign and native languages. Being replaced by another woman and losing dignity through the experience of intimate partner violence produced psychosomatic symptoms and mental health problems, such as feeling depressed, stressed and restless; sleeplessness; migraines; and stomach aches. Half of the women reported that their husbands had been unfaithful to them and become involved with other immigrant women from Thailand, Finland, the Philippines or Syria. Some of these men defended their actions by blaming the woman for the lack of erotic passion in the marriage or for not earning enough money or taking sufficient care of the family:

He had many women, but I never thought he could choose a new woman and throw me away. We had formed a business together selling fruit. He was my husband and business partner […] He was cheating [on me] and [brought] a new Filipino woman to Sweden. He hit me and hurt me. He said ‘you are getting old and are not desirable’ […]. I was homeless and depressed. (Kanda, 51, illiterate, entrepreneur)

Inevitably, in these infidelity situations, the older Thai wife was forced to move out of the house when another, often younger, the woman took her place. The interviewed women reported feeling stripped of their human dignity with no respect for their legal rights or any clear means of protecting their human rights or individual well-being.

### Broken dreams and deception

The interviewees perceived that the reality of life (for example, quarrels and conflicts concerning children from present and previous marriages) was definitely different from their dreams. These family difficulties sometimes involved an ex-wife or mother-in-law. The quarrels could start with an issue such as the Thai woman being expected to act as a nanny or servant to her husband’s children from a previous marriage or collecting them if they were coming home late. This type of extra service was never a concern in relation to Thai children; in those cases, the quarrels often concerned economics and the sharing of household costs. Moreover, some of the children witnessed violence and/or were maltreated themselves according to the women’s descriptions:

I divorced [in Thailand], my sister and her Swedish husband organised a guy for me; our goal was [to be] Mia farang, get a job and have a better life […]. Ever since he brought my little four-year-old daughter and me to Sweden, he maltreated my daughter: unfriendly, shouting at her, and ignorant; she was afraid of him. He was a jealous man; he did not allow me to send my daughter to bed and say ‘goodnight’. He requested that I be with him, not my daughter, 24 hours a day. We argued; he said he hated my daughter. We fought [physically], he hit me, pushed me and threw out my daughter and me. Luckily, my Swedish neighbour helped us before we got frostbitten and died in the snow […] the police came finally, I divorced him. (Keeta, 38, bachelor’s degree, factory worker and cleaner)

According to the Thai immigrant wives, their roles in family life often included being a maid, a housewife, a cleaner and a provider of sex. Contrary to the Thai women’s expectations, life in Sweden did not result in better status and rank, nor did they receive care and respect from their new husbands and families. Rather, the interviewed Thai women’s experiences were the opposite: they were often degraded and maltreated in their roles as Asian wives within the new family, but their neighbours and welfare services in the new country were helpful. Their dreams of being a Mia farang (Madam or Khun Nai), which included attaining a better and more comfortable life, did not come true. However, none of the interviewed women wanted to lose face and return home to Thailand, so they felt that they had to be patient with their husbands and partners and continue to strive for a better life in Sweden.

## Discussion

The present study explored Thai women’s experiences of intimate partner violence by Swedish men in international marriages. These Thai women described being faithful and silent and reliable housewives. However, this did not keep them from being replaced as wives and losing dignity due to IPV, resulting in broken dreams and deception from male partners and family in Sweden. Clearly, these Thai women’s international marriages were based on unequal power [28], and they lacked equality. The newly arrived Thai women ended up being dependent on their husbands, particularly because of the rule requiring a two-year residency before formal citizenship can be attained.

The present study found that Thai women presented themselves as faithful and silent, keeping the men’s intimate partner violence as a family issue. Norms, traditions and a lack of personal support and resources in the new country made the interviewed women endure their husbands’ violent acts. The women did not want to talk to friends or the authorities about the intimate partner violence they experienced, due to embarrassment and fear of being sent home and possibly due to previous marriage experiences that, for some, might have included abusive husbands. Previous research in Sweden found that Thai female immigrants had a rather high (22%) lifetime prevalence of IPV, although the prevalence was much lower (7%) in their current relationship [15].

The interviewed Thai women also brought up their husbands’ withdrawal from family obligations and responsibilities as related to the men’s intimate partner violence. The women’s explanations for the occurrence of violence were based on their husbands’ alcohol and drug problems, cultural differences (e.g. Western males not understanding Thai customs and traditions), immature behaviour by their partners, and infidelity on the husband’s behalf. Research findings support some of the women’s explanations; increased risk of IPV among female immigrants has previously been found to be related to males’ heavy alcohol consumption, poverty and lack of education [42–45], male psychopathology [27,46], and family dysfunction and intercultural differences [46–48]. At the individual level, research indicates that a combination of alcohol use, mental health problems and gender role expectations affects the relationship and, in turn, seems to reinforce and increase violence against women [49]. Broken dreams resulted when these international marriages did not meet imagined ideals; the interviewed women sometimes felt disrespected by Swedish family members, and they also reported psychosomatic symptoms and mental health problems based on their experiences of IPV.

It is understandable that Thai immigrant women have difficulties learning about their rights in Sweden [50], where to turn if they are victimised, and how to manage on their own if they decide to report their husband’s intimate partner violence. Most of the interviewees in the present study were educated, and only two were illiterate; some even had university education, although most worked as manual workers in Sweden, a finding that has also been reported by other researchers [26,51]. Previous research findings have shown that women who are empowered educationally, economically and socially are the most protected in situations of male intimate partner violence [19,20,46].

However, education does not seem to completely protect women from intimate partner violence by men; sometimes, education might provoke men to exert even more power [26], which seems to be the case for some of the imported wives in the present study. Education helps women to recognise their needs and rights, which might also be provoking to men. As Jewkes [52] argues, it is now time to include gender as well as socioeconomic inequalities as mainstream health considerations when teaching students and when discussing possible causes of ill health with professionals working in the area of health and welfare.

Moreover, according to the ecological model [30], men might react to women’s improvements and development of equity by resisting the social gender order and trying to maintain traditional views that include women’s subordination. Possibly, some Swedish men who import Thai women have reacted to social changes in Sweden involving women demanding equal rights with men by dreaming of a wife who is more adaptable to their needs and wants.

Finally, our findings and previous research [15] show that some Thai women are abused and maltreated by their partners in Sweden, and sometimes this maltreatment also includes children (see the above quotes by Chaba and Keeta). Women in Third World countries often see social acceptance of violence as a way of resolving family conflicts [30,31]. Although the interviewees in the present study were worried about their children’s safety, they often kept silent and patient as this was also the advice they received from Thai friends. The present study also found that disclosing family issues such as intimate partner violence is not an option, according to the interviewed Thai women. Moreover, as found in earlier research, receiving social support from Swedish friends is seldom possible [32].

Some Thai imported wives also found relations with their Swedish mothers-in-law and partner’s ex-wives very difficult, especially if previous Swedish children were included. Additionally, the female partners in the international marriages allowed these abusive relations to continue because being married to a Western man allowed them the possibility of starting a new life, even if a sexual relationship was the price of economic security [53–55]. In a broader, global context, imported wives can be viewed as a lightly disguised version of transnational trafficking, frequently referred to as a ‘modern form of slavery’ [56]. Women from Thailand who were trafficked into the Japanese sex industry during the 1980s and 1990s displayed the same general characteristics and motivation as the Thai women in this study, whose stated reasons for remaining in clearly abusive circumstances led to the same conclusions: in both cases, the Thai women were fighting to protect themselves and their children and to support their families at home [56].

## Methodological considerations

The methodological strengths of the present study are that all the interviews were conducted in the Thai participant’s language of choice (Thai, the Isan local language or Thai mixed with English). The interviewer in the present study was a native Thai speaker who could speak the necessary dialects and translate without risking misinterpretation. The use of a native Thai speaker also served to make the Thai participants feel comfortable, which was an important and delicate issue. As Lee et al. [56] noted, male intimate partner violence against women is a highly sensitive issue to investigate, and important details are often withheld by the interviewees due to embarrassment or humiliation.

The interviewer (WP) is a mental health nurse and nurse instructor with a master’s degree who at time of the interviews was a PhD student in nursing. WP is experienced in professional encounters with patients and nursing students, and at the time of the interviews she had studied in Sweden full-time for six years, since 2010. Additionally, having grown up in the same region as the interviewees, the interviewer understood the environmental influence of the Thai women’s traditions and cultural backgrounds.

## Limitations and difficulties

The limited number of women with experieces of IPV must be taken into consideration when discussing the saturation of data, but most women find it difficult to talk about this sensitive topic, irrespective of country of origin. This sample of immigrated women (so-called imported wives) is sufficient to claim saturation.

The interviewer found it tiring and sometimes difficult to handle negative feelings and thoughts related to the participants’ life circumstances and choices. As the interviewer asked for reflections on specific situations of everyday life and encouraged Thai women to explain and clarify, the interviews were in some cases heavily emotional . Several of these Thai immigrant women left their Thai children with family in Thailand, such as with grandparents or other relatives.

Future research needs to focus on the domestic reasons for Thai women’s migration and the cultural aspects and transnational responsibilities of imported wives. In addition, research needs to be conducted concerning the children involved – both those from previous marriages and those resulting from international marriages – and the negative effects that abusive relationships have on them.

## Conclusion

The vulnerability of imported wives in international marriages needs to be further recognised by health and welfare agencies in Sweden, as elsewhere, to ensure that these women have equal access to human rights, welfare and health as other citizens. From a health promotion perspective, home-based health check-ups are needed to stop the exploitation of imported wives. In Thailand, information and education about the unrecognised negative conditions of the Mia farang role need to be disseminated.

## Paper context

Sweden has 10 million inhabitants, and 16,000 of them are Thai women living with a Swedish man. The immigration of Thai women to Sweden for marriage has increased dramatically during the last years. Few research studies have focused on Thai women’s experiences of life in Sweden. This interview study focused on the lived experiences of domestic violence of Thai ‘imported wives’. Previous research employed quantitative measures to study IPV. Gender equity is not present in all Swedish marriages, especially those involving immigrant women, who experience a less equal situation, as shown in previous research.
